# Etiology-associated differences in the gut microbiome and metabolome in hepatocellular carcinoma: an exploratory study of alcohol-related and HBV-related cases

**DOI:** 10.3389/fmicb.2026.1927595

**Published:** 2026-09-30

**Authors:** Yongguang Wei, Kejian Yang, Qinchen Lu, Xin Zhou, Huasheng Huang, Meiliang Liu, Lijun Wang, Liming Shang, Tao Peng, Xiaoyun Zeng

**Affiliations:** 1 Department of Epidemiology and Health Statistics, School of Public Health, Guangxi Medical University, Nanning, Guangxi, China; 2 Department of Hepatobiliary Surgery, The First Affiliated Hospital of Guangxi Medical University, Nanning, China; 3 Guangxi Medical University, Nanning, China; 4 Guangxi Key Laboratory of Enhanced Recovery after Surgery for Gastrointestinal Cancer (Guangxi Medical University), Nanning, China; 5 Key Laboratory of Early Prevention and Treatment for Regional High Frequency Tumor (Guangxi Medical University), Ministry of Education, Nanning, Guangxi, China; 6 Department of Hepatobiliary Gland Surgery, The Ninth Affiliated Hospital of Guangxi Medical University, Beihai People’s Hospita, Beihai, Guangxi, China

**Keywords:** alcohol-related liver disease, gut microbiome, hepatitis B virus, hepatocellular carcinoma, metabolomics

## Abstract

Hepatocellular carcinoma (HCC) arises in heterogeneous etiological contexts, but direct cross-omics comparisons between HCC subgroups defined by different etiologies remain limited. In this exploratory study, we combined fecal 16S rRNA gene sequencing and untargeted serum metabolomics with exploratory cross-omics analyses to compare gut microbial composition, serum metabolic profiles, and microbiome–metabolite association patterns between hepatitis B virus-related HCC (HBV-HCC) and alcohol-related HCC (ALD-HCC). Forty patients who underwent primary tumor resection were included, comprising 26 with HBV-HCC and 14 with ALD-HCC. Alpha diversity indices did not differ significantly between groups, whereas within-group Bray–Curtis dissimilarity was greater in ALD-HCC and genus-level beta-diversity ordination showed only modest separation between the two groups. LEfSe-based screening suggested the enrichment of candidate taxa, including *Faecalibacterium*, *Roseburia*, *Lachnospira*, *Turicibacter*, and *Butyribacter* in HBV-HCC, whereas *Allisonella* and *Coprobacillus* were enriched in ALD-HCC. Untargeted metabolomics showed group separation in supervised models and identified exploratory candidate metabolic features mainly involving glycerophospholipid metabolism, primary bile acid biosynthesis, aminoacyl-tRNA biosynthesis, and central carbon metabolism in cancer; however, these metabolite-level findings did not remain significant after multiple-testing correction. Procrustes and O2PLS analyses indicated limited global concordance between the microbiome and metabolome, although exploratory associations were observed between several phosphatidylcholine-related features and microbial taxa. Overall, HBV-HCC and ALD-HCC may be associated with different gut microbial and serum metabolic patterns, and these findings should be considered exploratory and hypothesis-generating.

## Graphical abstract

**Figure:**
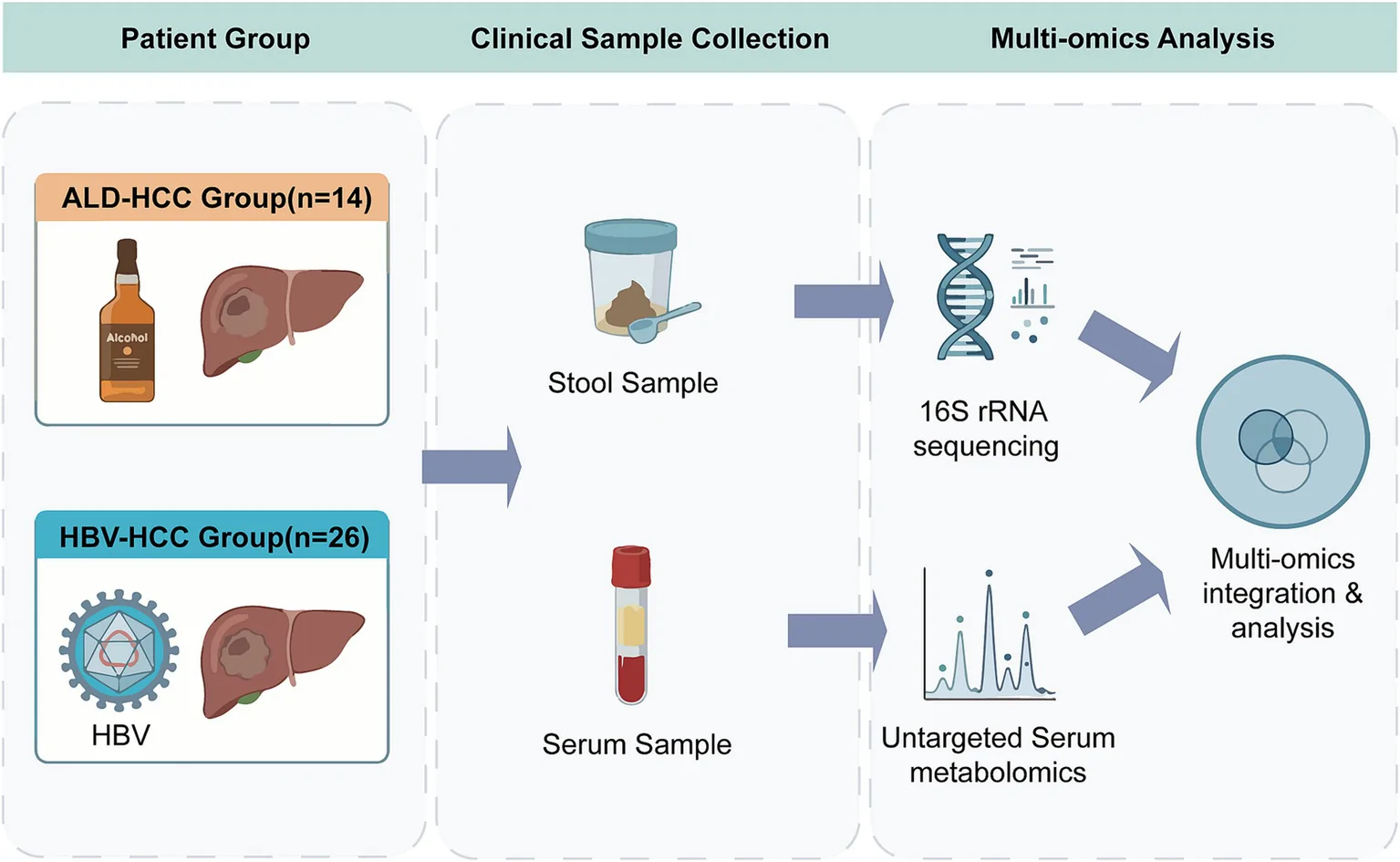

## 1 Introduction

Primary liver cancer remains a major global health burden, ranking sixth in incidence and third in cancer-related mortality worldwide (Sung et al., 2021). Hepatocellular carcinoma (HCC) is the predominant histological subtype and develops in the context of diverse risk factors, including chronic hepatitis B virus (HBV) infection, excessive alcohol consumption, metabolic dysfunction, and chronic inflammatory liver disease (Zhou et al., 2025; El–Serag and Rudolph, 2007; Huang et al., 2010). These exposures differ substantially in geographic distribution and biologic context, indicating that HCC should not be viewed as a uniform disease entity.

HBV-related and alcohol-related hepatocarcinogenesis involve partially distinct pathogenic mechanisms. HBV can promote malignant transformation through viral DNA integration, genomic instability, and epigenetic dysregulation (Kremsdorf et al., 2006; Hai et al., 2014), whereas chronic alcohol exposure is linked to acetaldehyde-mediated toxicity, oxidative stress, lipid peroxidation, inflammatory dysregulation, and altered hepatic metabolism (Taniai, 2020). Additional evidence suggests that viral hepatitis, metabolic dysfunction, and alcohol-related liver disease may be associated with distinct pathogenic pathways leading to HCC (Guerrero et al., 2025; Edoardo and Eleonora, 2024). These differences raise the possibility that HCC arising from different etiological backgrounds may also exhibit different host-microbial and metabolic features.

The gut-liver axis has become an important framework for understanding chronic liver disease and HCC (Hu et al., 2025; Luo et al., 2022). Owing to the portal circulation, the liver is continuously exposed to intestinal microbes and microbially derived products. Gut dysbiosis, barrier dysfunction, and altered microbial metabolite flux may contribute to chronic hepatic inflammation, immune dysregulation, and tumor-promoting microenvironments (Luo et al., 2022; Jinato et al., 2024; Jacob et al., 2023; Llorente et al., 2025). Both HBV infection and long-term alcohol exposure can disrupt intestinal homeostasis, but whether these distinct etiological contexts are associated with different microbiome configurations in established HCC remains incompletely understood.

Metabolic reprogramming is another central feature of HCC biology (Ward and Thompson, 2012). Serum metabolomics provides a complementary approach to characterizing host metabolic status and may capture perturbations in lipid, amino acid, bile acid, and carbon metabolism (Dunn et al., 2011; Casadei-Gardini et al., 2020; Wu et al., 2023). Because HBV infection and alcohol exposure are linked to different metabolic disturbances (Yan et al., 2025; Chen et al., 2022), metabolomic profiling may help clarify whether HCC arising from different etiologies is associated with distinct systemic metabolic patterns.

Most microbiome- and metabolome-based studies in HCC have focused on comparisons with healthy individuals or patients with cirrhosis, whereas direct comparisons between etiologically defined HCC subgroups remain relatively scarce (Jinato et al., 2024; Behary et al., 2021; Kim et al., 2019). Microbiome alterations have also been reported across the cirrhosis-to-HCC transition (Lapidot et al., 2020), and intratumoral bacterial profiles have been linked to metabolic alterations in HCC tissues (Xue et al., 2024). Recent studies have further highlighted diverse molecular and regulatory processes involved in HCC progression (Zhou et al., 2024; Ge et al., 2025; Xu et al., 2024), providing broader biological context for etiological heterogeneity in HCC. Against this background, we performed an exploratory comparison of gut microbial composition, serum metabolomic profiles, and microbiome–metabolite association patterns between HBV-HCC and ALD-HCC. Given the limited taxonomic resolution of 16S rRNA gene sequencing, the compositional nature of relative-abundance microbiome data, and the indirect functional inference provided by PICRUSt2, these analyses were interpreted as exploratory rather than mechanistic. The study therefore aimed to identify etiology-associated microbial and metabolic patterns that may inform future validation and mechanistic investigations.

## 2 Materials and methods

### 2.1 Clinical subjects

This study was approved by the Medical Ethics Committee of the First Affiliated Hospital of Guangxi Medical University (NO.2023-S960-01). Participant enrollment and sample collection commenced after ethics approval. Patients with newly diagnosed HCC who underwent primary tumor resection at the First Affiliated Hospital of Guangxi Medical University were consecutively enrolled from December 2023 to May 2024. A total of 40 patients were included, comprising 14 with ALD-HCC and 26 with HBV-HCC. All participants provided written informed consent for participation and use of their samples. HCC was diagnosed according to contemporary Chinese clinical guidelines (Zhou et al., 2025), and patients were classified into mutually exclusive ALD-HCC and HBV-HCC groups according to prespecified etiologic criteria.

ALD-HCC was defined by a long-term history of alcohol exposure, generally >5 years, with alcohol intake >40 g/day for men or >20 g/day for women, consistent with Chinese guidance for alcoholic liver disease (National Workshop on Fatty Liver and Alcoholic Liver Disease CSoH, Chinese Medical Association et al., 2018), together with HBV negativity. HBV-HCC was defined by hepatitis B surface antigen positivity, no prior antiviral therapy, and no alcohol exposure meeting the above threshold. Patients with mixed etiologies were excluded. Additional exclusion criteria included prior targeted therapy, immunotherapy, or interventional treatment before sampling; other malignancies; and use of antibiotics, probiotics, prebiotics, or gastrointestinal motility drugs within 1 month before sample collection.

### 2.2 Sample collection

Fecal samples were collected immediately after spontaneous defecation, and the midstream portion was transferred into sterile 50-mL centrifuge tubes and stored at −80 °C immediately after collection. Fasting blood samples were obtained in the morning and collected into anticoagulant-free tubes. After clotting at room temperature for 30 min, samples were centrifuged at 4 °C and 1,500 x g for 10 min. The separated serum was aliquoted into 200-uL tubes to avoid repeated freeze–thaw cycles and stored at −80 °C until further analysis.

### 2.3 16S rRNA gene sequencing and microbiome analysis

Fecal 16S rRNA gene sequencing was performed by AIage Life Science Corporation Ltd. (Nanning, China). The V3–V4 hypervariable region of the bacterial 16S rRNA gene was amplified using primers 341F (5′-CCTACGGGNGGCWGCAG-3′) and 806R (5′-GACTACHVGGGTATCTAATCC-3′) and sequenced on the Illumina MiSeq platform. Sequence data were processed using QIIME 2 v2024.10 with DADA2 to generate amplicon sequence variants (ASVs) (Callahan et al., 2016). Taxonomy was assigned using the QIIME 2 classify-sklearn method with a pretrained naive Bayes classifier based on the SILVA 138.2 bacterial reference database at a confidence threshold of 0.7 (Bolyen et al., 2019). After denoising and chimera removal, 11,907–41,911 sequences were retained per sample.

For alpha- and beta-diversity analyses, the ASV table was rarefied to 11,907 sequences per sample, corresponding to the minimum sequencing depth after denoising and chimera removal across all samples. Bray–Curtis dissimilarity was used for beta-diversity analysis, and between-group differences were assessed by PERMANOVA with 999 permutations. Differential taxa were screened using LEfSe with the all-against-all strategy. Because only two classes, ALD-HCC and HBV-HCC, were included, this corresponded to a single between-group comparison. LEfSe applied the Kruskal–Wallis test followed by an unpaired Wilcoxon rank-sum test, with linear discriminant analysis used to estimate effect sizes. Taxa with nominal *p* < 0.05 and an LDA score >2 were considered candidate discriminative taxa (Segata et al., 2011). A genus-level ANCOM-BC2 analysis based on raw count data and adjusted for age, BMI, diabetes, and hypertension was additionally performed as a composition-aware sensitivity analysis. Multivariate dispersion was assessed using a median-based betadisper analysis with 999 permutations.

Predicted microbial functional profiles were generated using PICRUSt2 v2.2.0 at the individual-sample level without within-group pooling (Douglas et al., 2020). KEGG Level 3 pathways were ranked by total predicted functional abundance across all samples, and the 30 most abundant pathways were displayed as sample-wise relative abundances using a logarithmic color scale. This Top-30 criterion was used only for visualization; no between-group differential-function test or pathway-level significance threshold was applied. PICRUSt2 results were therefore interpreted as exploratory functional predictions rather than experimentally validated functional differences.

### 2.4 Untargeted metabolomic analysis

Serum samples were subjected to untargeted metabolomic profiling by Majorbio Bio-Pharm Technology Co., Ltd. (Shanghai, China). Metabolites were separated on an HSS T3 column (100 mm × 2.1 mm, 1.8 μm) using water/acetonitrile (95/5, v/v) containing 0.1% formic acid as solvent A and acetonitrile/isopropanol/water (47.5/47.5/5, v/v/v) containing 0.1% formic acid as solvent B. Data were acquired on a UHPLC-Q Exactive HF-X mass spectrometry platform (Thermo Fisher Scientific, United States) in both positive and negative ion modes.

Raw LC–MS data were processed using Progenesis QI for baseline filtering, peak detection and integration, retention-time correction, and peak alignment. Within each ion mode, a prespecified 80% detection-rate threshold reduced the initial 27,717 ion features to 18,656. Remaining missing values were handled by minimum-value imputation, followed by sum normalization within each ion mode. Features with a QC relative standard deviation >30% were then excluded, yielding 14,287 features, after which the data were log10-transformed for downstream analysis.

Metabolite annotation was performed by matching acquired MS and MS/MS information against the service provider’s in-house spectral library and public or theoretical databases, including HMDB and METLIN, using an MS mass-error criterion of <10 ppm. Annotation confidence was reported using the service provider’s specific B(i)/B(ii) scheme. B(i) annotations were based on matching experimental sample MS/MS spectra against the provider’s standard-derived in-house spectral library, whereas B(ii) annotations were based on matching against computationally simulated MS/MS libraries from public or theoretical databases. These categories were not mapped to formal MSI levels, and B(i) did not represent confirmation using project-specific authentic standards. PLS-DA and OPLS-DA were performed using ropls (version 1.6.2). KEGG compound classification and pathway mapping were based on KEGG kegg_v20230830, and pathway enrichment analysis was performed using scipy (version 1.0.0) with nominal *p* < 0.05.

### 2.5 Statistical analysis

Baseline clinical analyses were performed using SPSS 19.0. Normally distributed continuous variables were presented as mean ± SD and were compared using Student’s *t*-test, whereas non-normally distributed variables were presented as median [IQR] and were compared using the Wilcoxon rank-sum test. Categorical variables were presented as counts and percentages and were compared using Pearson’s chi-square test. Unless otherwise specified, all tests were two-sided and nominal *p* < 0.05 was considered statistically significant.

Exploratory candidate metabolic features were screened using nominal *p* < 0.05 and OPLS-DA VIP > 1, with fold change used only to indicate the direction of differences. Sensitivity analyses were performed to address clinical confounding, compositionality, and multiple testing. Metabolomic features were adjusted for etiology, age, BMI, diabetes, and hypertension, with Benjamini–Hochberg correction applied to etiology-effect tests. For microbiome–metabolite associations, raw ASV counts were aggregated to genus level, a fixed pseudocount of 0.5 was added before centered log-ratio transformation, and two-sided Spearman correlations were recalculated with BH correction across the predefined associations.

## 3 Results

### 3.1 Baseline characteristics of the study population

A total of 40 patients with HCC were included, comprising 14 patients with ALD-HCC and 26 with HBV-HCC (Table 1). All enrolled participants were male. Patients in the HBV-HCC group were significantly younger at diagnosis than those in the ALD-HCC group (Table 1, *p* < 0.05). A higher proportion of patients in the ALD-HCC group had a history of hypertension or diabetes mellitus (Table 1, *p* < 0.05). No significant differences were found in other demographic or clinical characteristics, including ethnicity, body mass index (BMI), liver fluke infection, Performance Status (PS) score, total bilirubin, albumin, prothrombin time, alpha-fetoprotein (AFP), protein induced by vitamin K absence (PIVKA-II), cirrhosis, splenomegaly, fatty liver disease, China Liver Cancer (CNLC) stage, or Barcelona Clinic Liver Cancer (BCLC) stage (Table 1, *p* > 0.05).

**Table 1:** Baseline characteristics of patients with hepatocellular carcinoma.

Variable	Level	Overall	ALD-HCC	HBV-HCC	*P*
*N*		40	14	26	
Age (years; median[IQR])		55.0[45.8, 62.2]	59.0 [52.0, 67.8]	53.5 [42.2, 59.0]	0.041
Gender	Male	40 (100.0)	14 (100.0)	26 (100.0)	NA
Ethnicity	Han Chinese	19 (47.5)	5 (35.7)	14 (53.8)	0.082
	Yao Chinese	4 (10.0)	0 (0.0)	4 (15.4)	
	Zhuang Chinese	17 (42.5)	9 (64.3)	8 (30.8)	
BMI (kg/m^2^; median[IQR])		24.0[20.7, 26.7]	23.5 [19.6, 26.7]	24.2 [21.0, 26.5]	0.671
Hypertension	No	30 (75.0)	7 (50.0)	23 (88.5)	0.018
	Yes	10 (25.0)	7 (50.0)	3 (11.5)	
Diabetes	No	33 (82.5)	9 (64.3)	24 (92.3)	0.039
	Yes	7 (17.5)	5 (35.7)	2 (7.7)	
Liver fluke infection	No	31 (77.5)	10 (71.4)	21 (80.8)	0.694
	Yes	9 (22.5)	4 (28.6)	5 (19.2)	
PS	0	34 (85.0)	11 (78.6)	23 (88.5)	0.646
	1	6 (15.0)	3 (21.4)	3 (11.5)	
Child-pugh	A	40 (100.0)	14 (100.0)	26 (100.0)	NA
TBiL (μmol/l; median [IQR])		10.1 [6.9, 16.7]	8.3 [6.0, 11.1]	11.2 [7.6, 17.2]	0.133
ALB (g/l; median [IQR])		40.3 [37.1, 42.0]	40.3 [38.4, 41.0]	40.0 [36.9, 42.3]	0.887
PT (s; median [IQR])		11.3 [10.8, 12.0]	11.1 [10.8, 11.7]	11.4 [10.9, 12.1]	0.435
AFP (ng/ml; median [IQR])		46.9 [6.2, 748.0]	9.2 [3.7, 73.5]	208.5 [10.8, 1433.4]	0.079
PIVKA-II (mAU/ml; median [IQR])		489.5 [116.0, 1331.5]	383.5 [56.6, 1860.8]	489.5 [144.8, 1159.8]	0.777
Cirrhosis	No	22 (55.0)	9 (64.3)	13 (50.0)	0.510
	Yes	18 (45.0)	5 (35.7)	13 (50.0)	
Splenomegaly	No	33 (82.5)	11 (78.6)	22 (84.6)	0.679
	Yes	7 (17.5)	3 (21.4)	4 (15.4)	
FLD	No	34 (85.0)	12 (85.7)	22 (84.6)	1.000
	Yes	6 (15.0)	2 (14.3)	4 (15.4)	
CNLC stage	Ia	23 (57.5)	9 (64.3)	14 (53.8)	0.816
	Ib	10 (25.0)	3 (21.4)	7 (26.9)	
	IIa	5 (12.5)	1 (7.1)	4 (15.4)	
	IIIa	2 (5.0)	1 (7.1)	1 (3.8)	
BCLC stage	0	3 (7.5)	1 (7.1)	2 (7.7)	0.922
	A	30 (75.0)	11 (78.6)	19 (73.1)	
	B	5 (12.5)	1 (7.1)	4 (15.4)	
	C	2 (5.0)	1 (7.1)	1 (3.8)	

ALD-HCC, alcohol-related hepatocellular carcinoma; HBV-HCC, hepatitis B virus-related hepatocellular carcinoma; PS, Performance Status; TBiL, total bilirubin; ALB, albumin; PT, prothrombin time; AFP, alpha-fetoprotein; PIVKA-II, protein induced by vitamin K absence; FLD, fatty liver disease.

### 3.2 Gut microbial diversity and community structure

We performed 16S rRNA gene sequencing on fecal samples from all 40 patients. The species-accumulation and Shannon rarefaction curves approached a plateau before the common rarefaction depth of 11,907 sequences per sample (Figure 1A and Supplementary Figure S1A), supporting adequate coverage of the major observed microbial diversity. Sequencing summary metrics are provided in Supplementary Table S1. Alpha diversity did not differ significantly between groups. Although the ALD-HCC group showed lower Sobs, Chao1, ACE, and Shannon indices and higher Simpson and coverage values than the HBV-HCC group, none of these differences reached statistical significance (all *p* > 0.05; Figures 1B,D–I and Supplementary Figure S1B).

**Figure 1:**
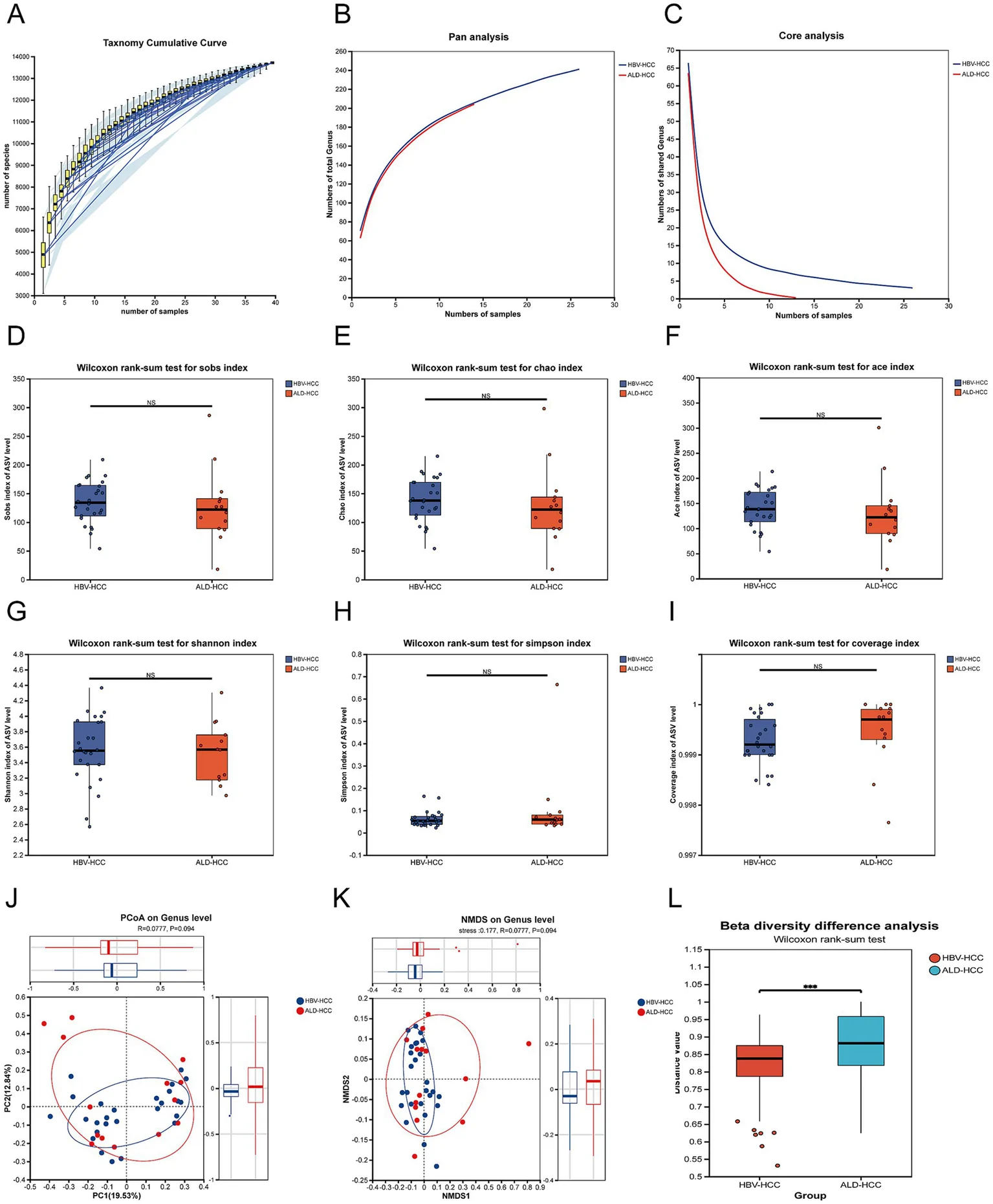
Gut microbial diversity based on 16S rRNA gene sequencing. **(A)** Taxonomic accumulation curve. **(B)** Pan analysis. **(C)** Core analysis. **(D–I)** Alpha-diversity indices. **(J)** Principal coordinates analysis (PCoA) based on Bray–Curtis dissimilarity. **(K)** Non-metric multidimensional scaling (NMDS) based on Bray–Curtis dissimilarity. **(L)** Between-group comparison of beta-diversity dissimilarity. Group comparisons in panels **(D–I,L)** were performed using the Wilcoxon rank-sum test. ^ns^*p* > 0.05; *p < 0.05, **p < 0.01, ***p < 0.001.

At the genus level, PCoA and NMDS showed only modest separation between HBV-HCC and ALD-HCC, with substantial overlap between groups (Figures 1J,K; R = 0.0777, *p* = 0.094). Within-group dissimilarity values were higher in ALD-HCC than in HBV-HCC (Figures 1C,L; *p* < 0.001), suggesting greater descriptive heterogeneity within the ALD-HCC group. However, PERMDISP showed no significant difference in multivariate dispersion between groups (*F* = 1.4145, *p* = 0.253).

### 3.3 Gut microbiota composition in ALD-HCC and HBV-HCC

At the genus level, 241 genera were identified in HBV-HCC and 204 in ALD-HCC, with 174 shared genera (Figure 2A). In both groups, the gut microbial community was dominated by *Blautia* and *Bacteroides* (Figure 2B). The Gut Microbiome Health Index (GMHI), a microbiome-derived index intended to summarize health-associated community structure (Gupta et al., 2020), was significantly lower in ALD-HCC than in HBV-HCC (Figure 2D, *P* < 0.001). In addition, GMHI showed a positive correlation with the Shannon index (Figure 2C, Spearman *r* = 0.31, *p* = 0.05).

**Figure 2:**
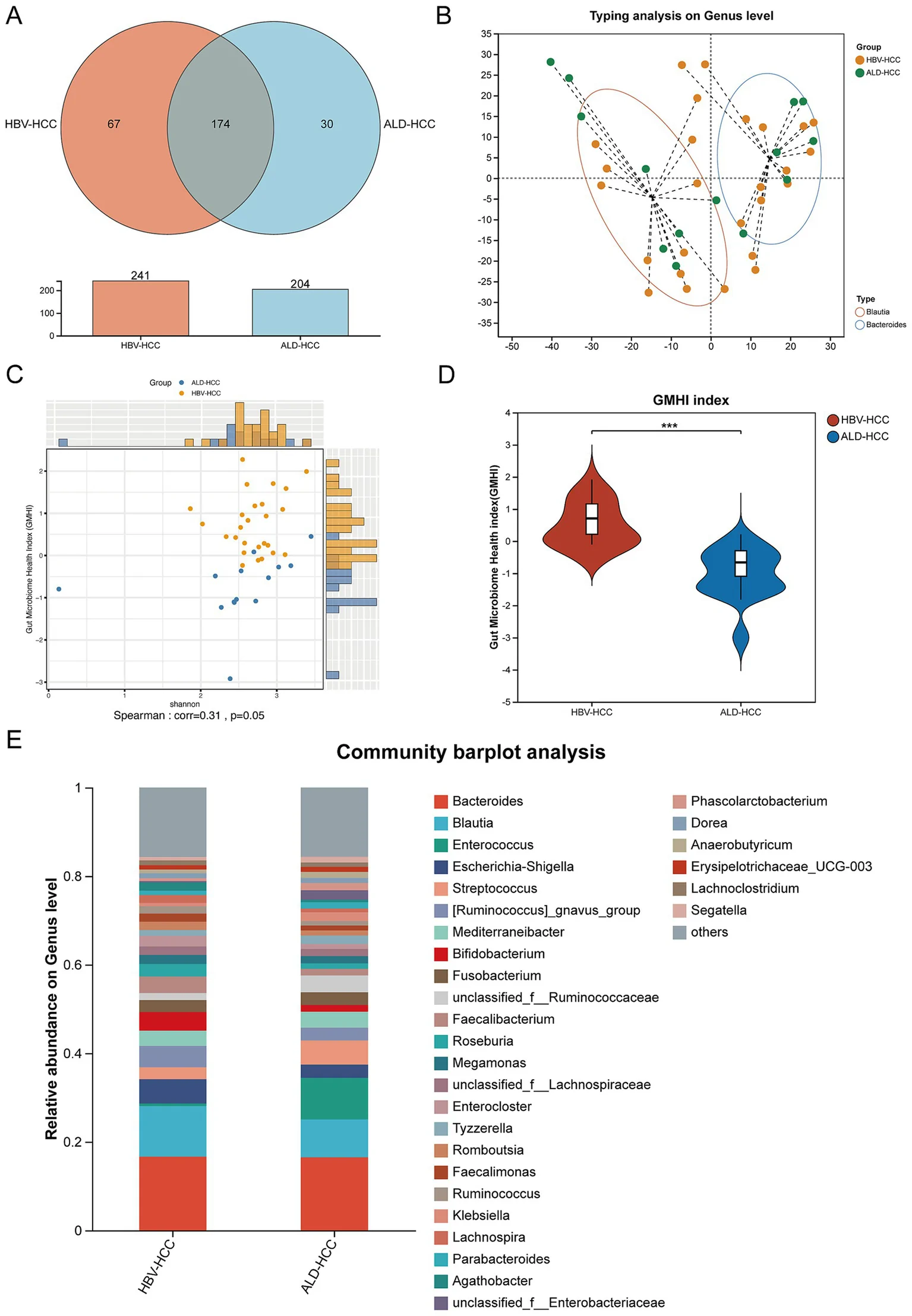
Taxonomic composition and microbial health index analysis. **(A)** Genus-level Venn diagram comparison. **(B)** Genus-level microbial classification map. **(C)** Correlation analysis between the Gut Microbiome Health Index (GMHI) and the Shannon index. **(D)** GMHI violin plot. **(E)** Genus-level community bar plot. Statistical significance was determined using the Wilcoxon rank-sum test. ^ns^*p* > 0.05, *p < 0.05, **p < 0.01, ***p < 0.001.

At the phylum level, the five most abundant taxa were Bacillota, Bacteroidota, Pseudomonadota, Actinomycetota, and Fusobacteriota in both groups (Supplementary Figure S2A). At the genus level, the dominant taxa included *Bacteroides*, *Blautia*, *Enterococcus*, *Escherichia-Shigella*, *Streptococcus*, *Ruminococcus gnavus* group, *Mediterraneibacter*, *Bifidobacterium*, *Fusobacterium*, and unclassified *Ruminococcaceae* (Figure 2E).

### 3.4 Differential microbial taxa and functional prediction

LEfSe-based screening identified candidate microbial features across taxonomic levels between the two groups (Figures 3A,B). At the genus level, *Faecalibacterium*, *Roseburia*, *Lachnospira*, *Turicibacter*, and *Butyribacter* showed candidate enrichment in HBV-HCC, whereas *Allisonella* and *Coprobacillus* were enriched in ALD-HCC (Figures 3C–J). Additional higher-level candidate taxa enriched in ALD-HCC included Lactobacillales, *Lactobacillaceae*, and *Succinivibrionaceae*. The covariate-adjusted ANCOM-BC2 sensitivity analysis provided partial support for the original LEfSe findings. Among 102 tested genera, 25 remained significant after BH correction (Supplementary Table S3). All four directly comparable core LEfSe genera showed concordant effect directions, with *Turicibacter* and *Lachnospira* remaining FDR-significant, whereas *Faecalibacterium* and *Roseburia* showed directionally consistent but non-significant associations after multiple-testing correction.

**Figure 3:**
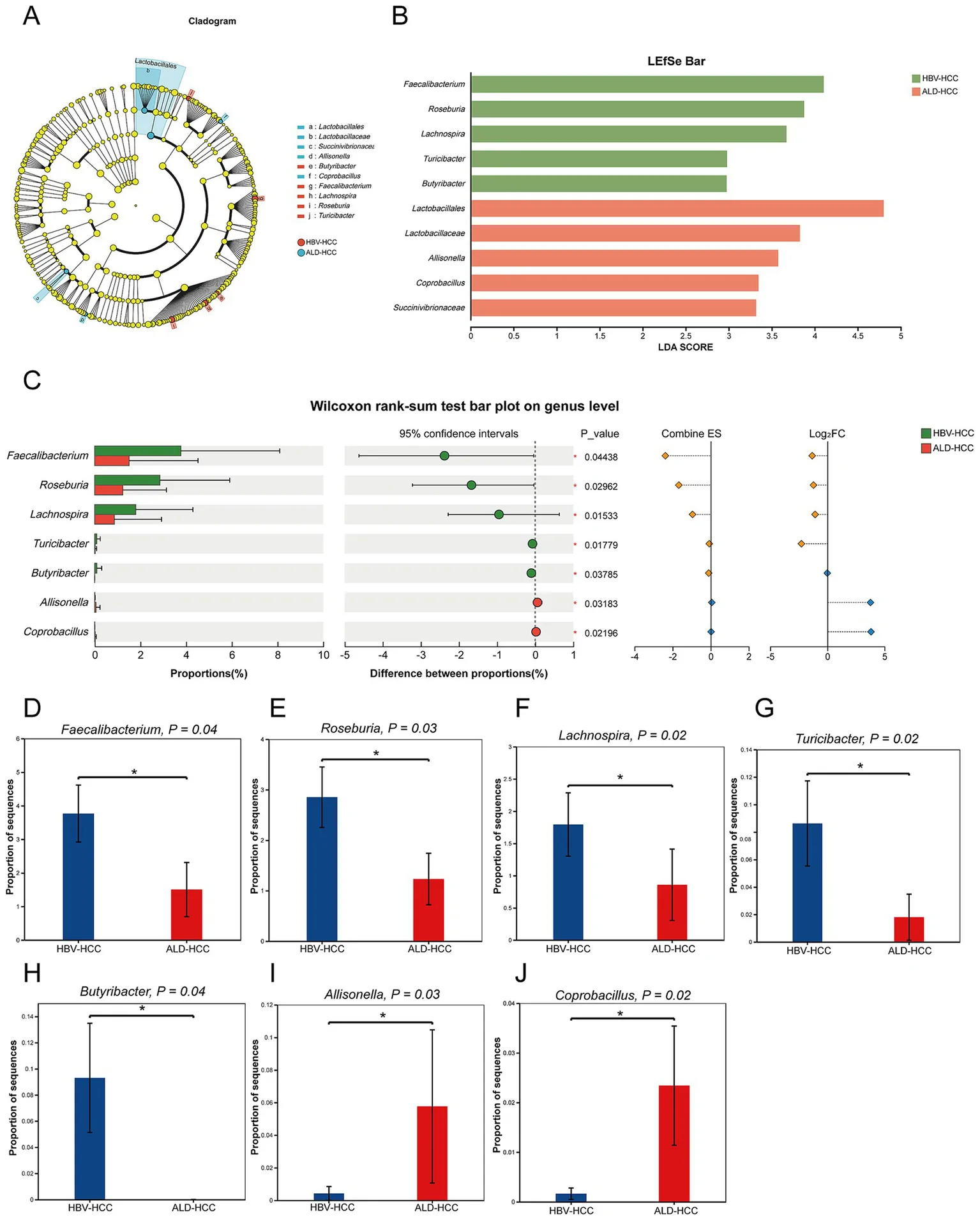
Exploratory screening of candidate microbial taxa between the ALD-HCC and HBV-HCC groups. **(A)** LEfSe cladogram. **(B)** LDA score bar plot. **(C)** Genus-level differential abundance comparison. **(D–J)** Genus-level abundance plots for *Faecalibacterium*, *Roseburia*, *Lachnospira*, *Turicibacter*, *Butyribacter*, *Allisonella*, and *Coprobacillus*. LEfSe was performed using the all-against-all strategy, with an LDA score > 2 and nominal *p* < 0.05 as the screening thresholds. ^ns^*p* > 0.05, *p < 0.05, **p < 0.01, ***p < 0.001.

PICRUSt2-based functional prediction indicated that both groups were dominated by broad metabolic and housekeeping functions, including metabolic pathways, biosynthesis of secondary metabolites, amino acid biosynthesis, ABC transporters, carbon metabolism, and ribosome-related functions (Figure 4A). COG classification showed a similar pattern, with amino acid and carbohydrate transport and metabolism, translation and ribosomal biogenesis, and transcription among the predominant predicted functions (Figures 4B,C). No between-group differential-function test was performed; therefore, these profiles were interpreted descriptively as predicted functional patterns rather than statistically validated functional differences.

**Figure 4:**
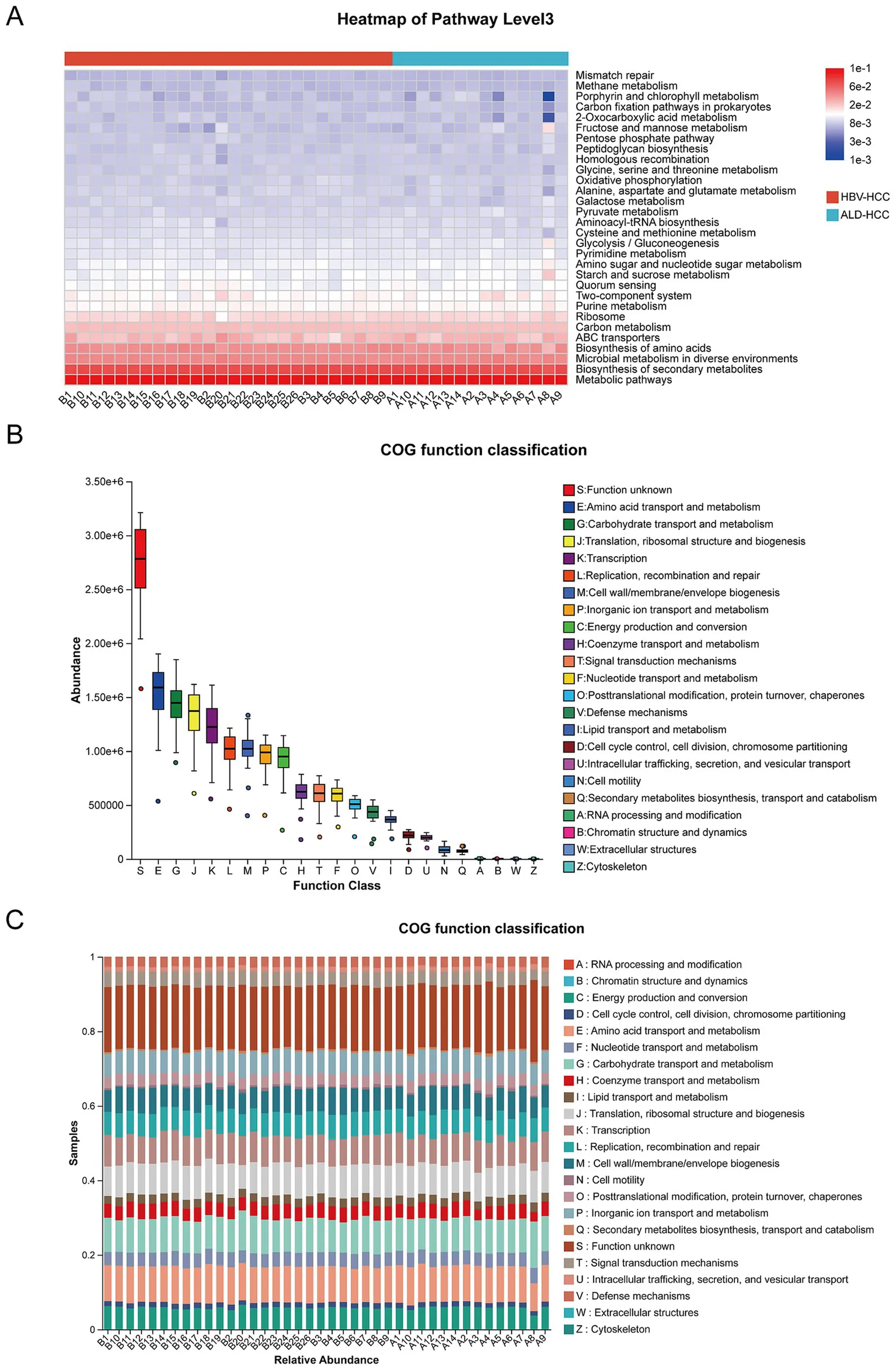
PICRUSt2-based functional prediction of the gut microbiota in ALD-HCC and HBV-HCC. **(A)** The 30 KEGG Level 3 pathways with the highest total predicted abundance across all samples. **(B)** COG functional prediction. **(C)** COG functional classification.

### 3.5 Serum metabolomic overview

Untargeted serum metabolomic profiling was performed in the HBV-HCC and ALD-HCC groups. Quality-control assessment indicated acceptable analytical reproducibility, with the cumulative proportion of features meeting the 30% RSD criterion exceeding 0.7 after preprocessing (Figure 5A). Additional QC and sample-correlation results are shown in Supplementary Figure S3, and feature counts before and after preprocessing are summarized in Supplementary Table S2. In the combined positive- and negative-ion data, 2,482 features were detected in ALD-HCC and 2,526 in HBV-HCC, of which 2,422 were shared between groups (Figure 5B).

**Figure 5:**
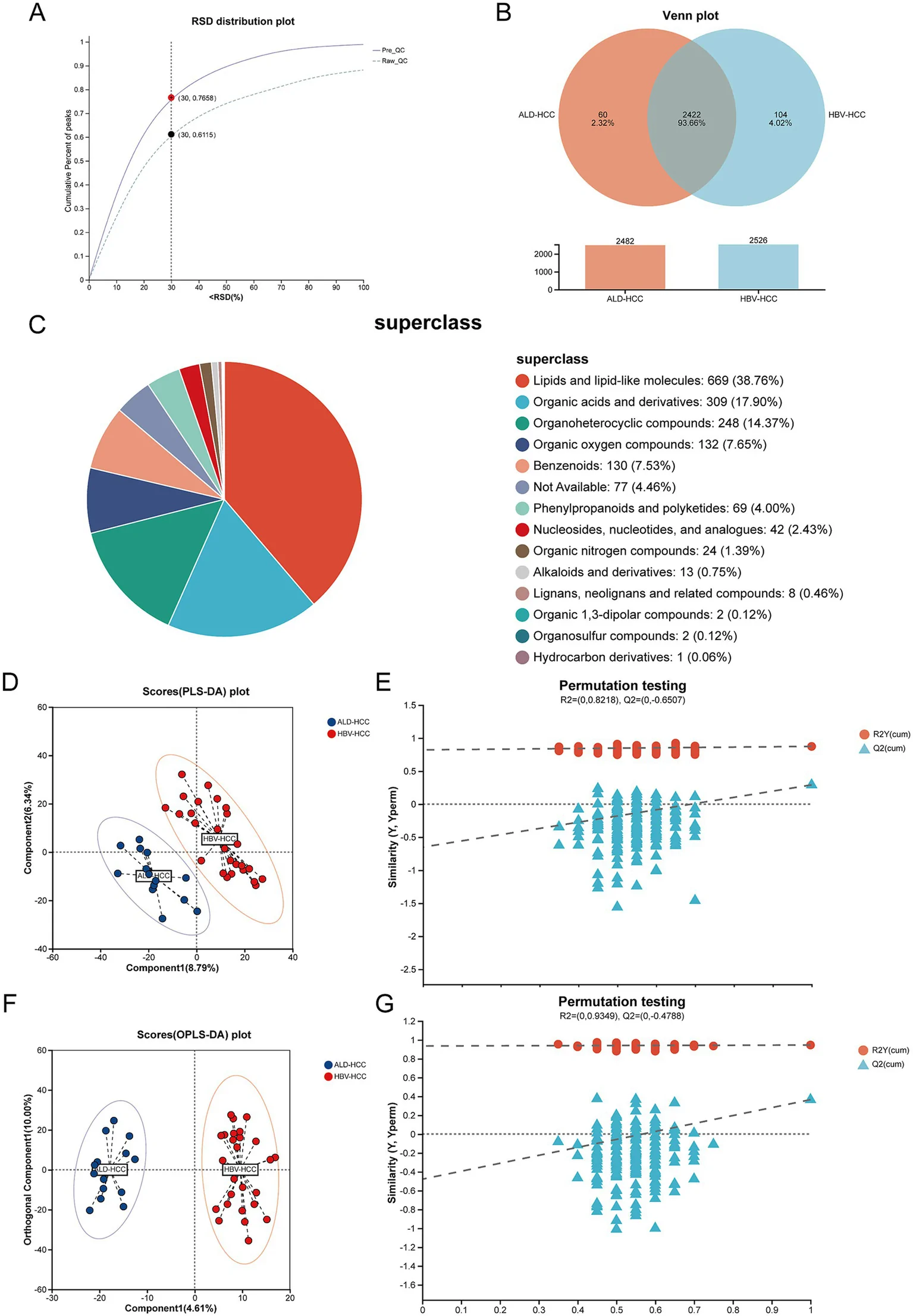
Metabolomics analysis between HBV-HCC and ALD-HCC groups. **(A)** QC sample evaluation plot. **(B)** Metabolic-feature Venn diagram. **(C)** HMDB superclass classification pie chart. **(D)** PLS-DA score scatter plot. **(E)** PLS-DA permutation test plot. **(F)** OPLS-DA score scatter plot. **(G)** OPLS-DA permutation test plot.

Among putatively annotated metabolic features, HMDB superclass distributions were broadly similar between groups and were dominated by lipids and lipid-like molecules, organic acids and derivatives, organoheterocyclic compounds, organic oxygen compounds, and benzenoids (Figure 5C). Supervised PLS-DA and OPLS-DA showed separation between HBV-HCC and ALD-HCC (Figures 5D–G).

### 3.6 Exploratory candidate metabolic features and pathway annotation

Using the prespecified exploratory criteria of nominal *p* < 0.05 and OPLS-DA VIP > 1, 1,653 ion features were identified, of which 221 were putatively annotated and constituted the exploratory candidate set used for downstream descriptive and pathway analyses. Among these 221 candidates, 29 showed higher and 192 lower abundance in ALD-HCC relative to HBV-HCC (Figures 6A–C). None of the 221 putatively annotated candidates remained significant after BH correction. In the covariate-adjusted sensitivity analysis accounting for age, BMI, diabetes, and hypertension, all 221 retained their original effect directions, but only 79 remained nominally significant and none reached BH-adjusted *q* < 0.05. Accordingly, these features were retained only as exploratory, hypothesis-generating candidates rather than FDR-supported differential metabolites.

**Figure 6:**
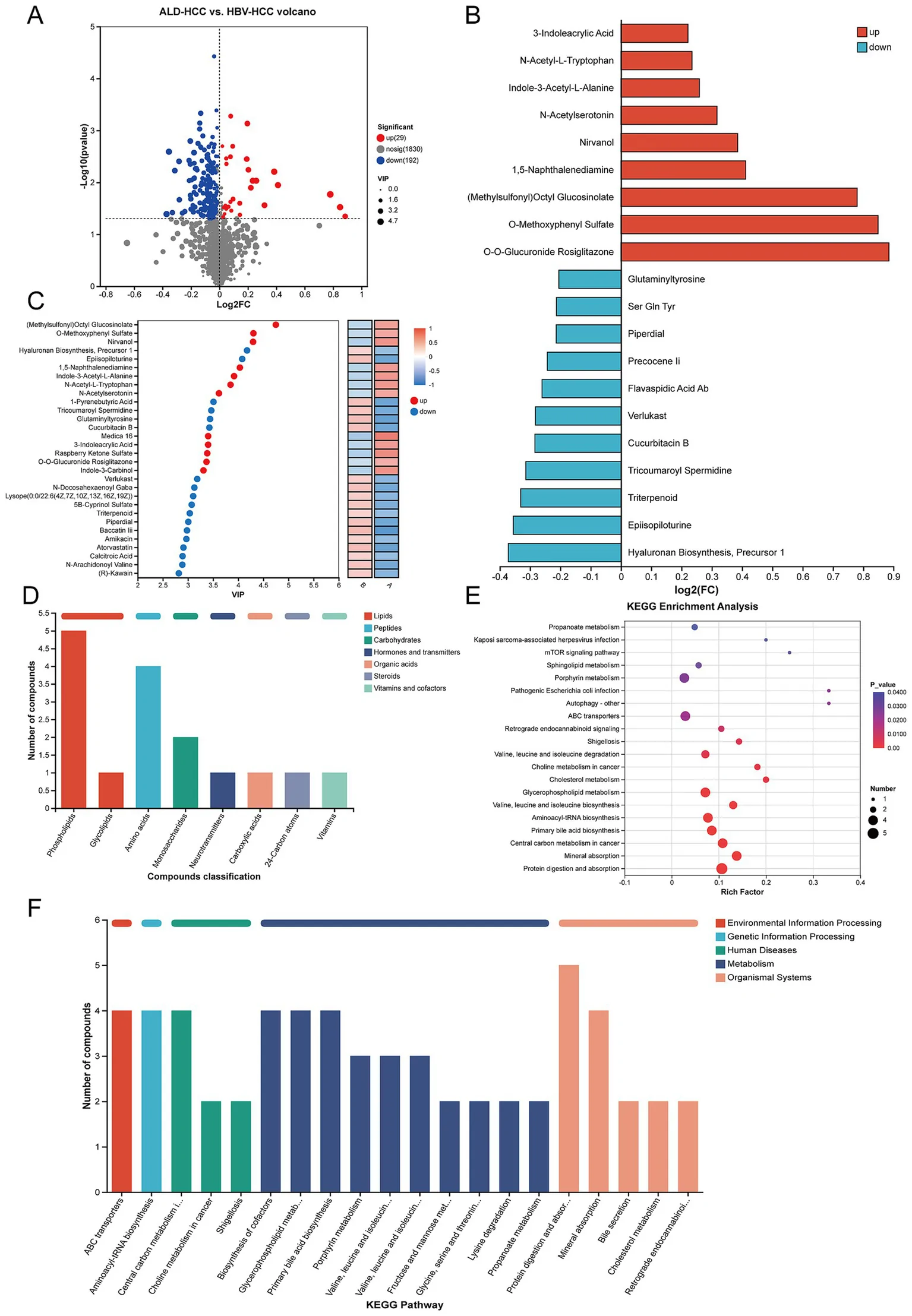
Identification and functional annotation of differential metabolites between ALD-HCC and HBV-HCC groups. **(A)** Volcano plot of differential metabolites. **(B)** Fold-change bar plot of differential metabolites. **(C)** VIP bubble plot of differential metabolites. **(D)** KEGG compound classification of differential metabolites. **(E)** KEGG pathway enrichment bubble plot of differential metabolites. **(F)** KEGG Level 3 functional annotation of differential metabolites.

The KEGG compound classification showed that these candidates were mainly assigned to phospholipids, amino acids, monosaccharides, glycolipids, neurotransmitters, carboxylic acids, 24-carbon compounds, and vitamins (Figure 6D). Exploratory pathway enrichment highlighted ABC transporters, aminoacyl-tRNA biosynthesis, central carbon metabolism in cancer, biosynthesis of cofactors, glycerophospholipid metabolism, primary bile acid biosynthesis, protein digestion and absorption, and mineral absorption (Figures 6E,F).

### 3.7 Microbiome–metabolite association analysis

Global cross-omics analyses indicated limited concordance between the microbiome and metabolome. Procrustes analysis showed poor agreement between the two data structures (*M*^2^ = 0.931, *p* = 0.255), and O2PLS likewise suggested only modest shared variation between the two omics layers (Figures 7A–C). Group-specific O2PLS results for ALD-HCC and HBV-HCC are presented in Supplementary Figures S4, S5, respectively.

**Figure 7:**
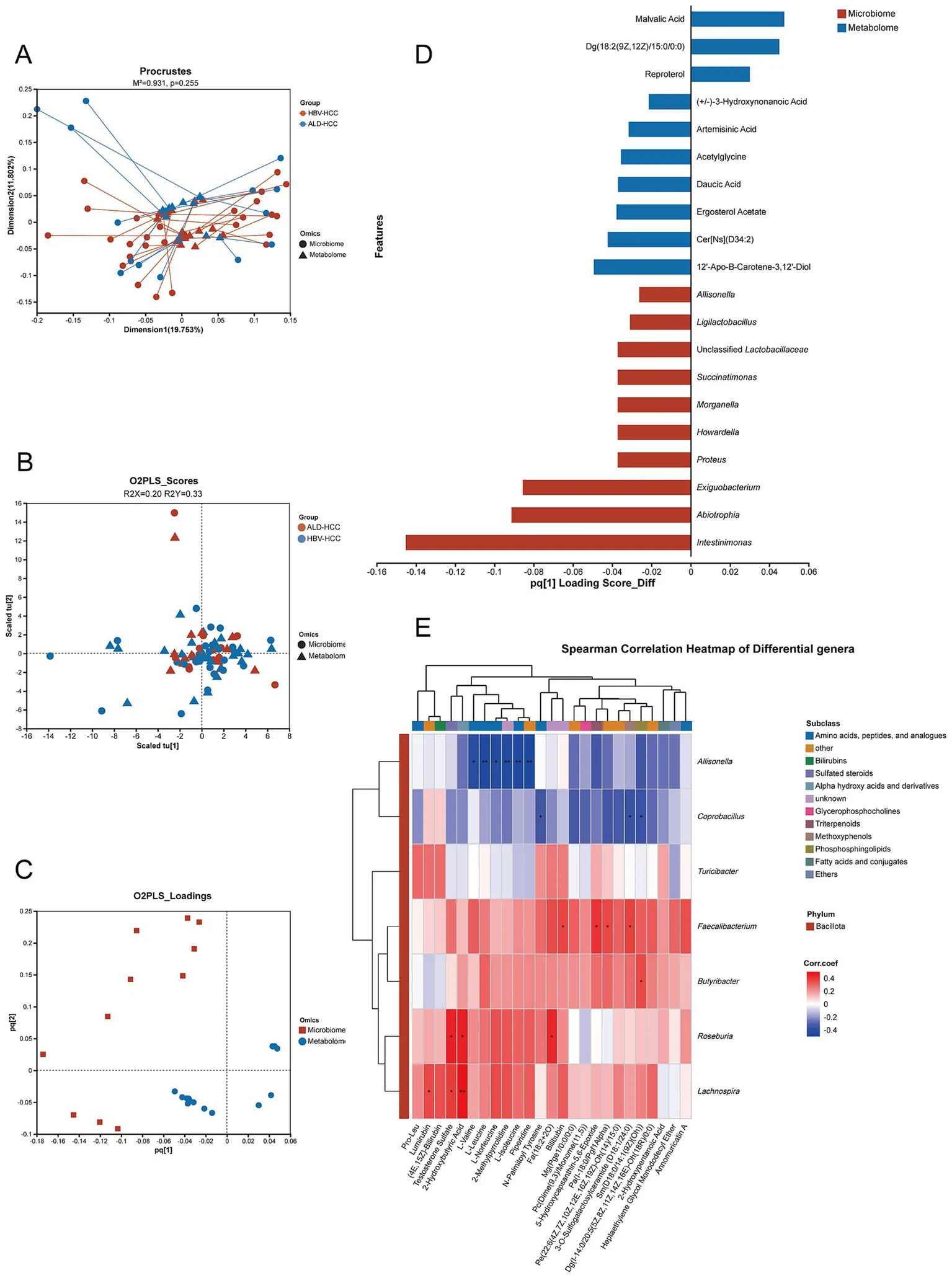
Correlation and integration analysis between the gut microbiota and metabolome. **(A)** Procrustes analysis of gut microbiota and metabolites. **(B)** O2PLS joint score plot. **(C)** O2PLS loading distribution plot. **(D)** Top 10 loading species and metabolites distribution plot. **(E)** Correlation heatmap in the differential feature set. Statistical significance was determined using Spearman correlation analysis. ^ns^*p* > 0.05, *p < 0.05, **p < 0.01, ***p < 0.001.

Exploratory correlation analyses identified associations between several putatively annotated metabolic features and microbial taxa (Figures 7D,E). Phosphatidylcholine-related features showed recurrent associations with multiple taxa in both HBV-HCC and ALD-HCC (Supplementary Figures S4D, S5D), while candidate taxa including *Lachnospira*, *Roseburia*, *Faecalibacterium*, *Allisonella*, and *Coprobacillus* were associated with selected metabolic features in the combined analysis. In the CLR-based sensitivity analysis, 32 of the 34 core relationships retained their original direction; however, none of the 1,425 tested associations remained significant after BH correction. These correlation patterns were therefore retained only as exploratory, hypothesis-generating associations rather than evidence of a robust microbiome–metabolite interaction network or directional regulation.

## 4 Discussion

In this exploratory study, we identified distinct gut microbial and serum metabolic patterns associated with HBV-HCC and ALD-HCC. Direct comparisons between etiologic subgroups within established HCC remain relatively uncommon, as most previous microbiome and metabolome studies have focused on comparisons between HCC and healthy individuals, cirrhosis, or other non-HCC liver diseases (Behary et al., 2021; Kim et al., 2019; Lapidot et al., 2020). HBV-HCC and ALD-HCC also arise against different biological backgrounds: HBV-related hepatocarcinogenesis is closely linked to viral integration and HBV-associated genomic and epigenetic dysregulation, whereas alcohol-related hepatocarcinogenesis is more strongly associated with acetaldehyde toxicity, oxidative stress, lipid peroxidation, and chronic inflammatory-metabolic injury (Guerrero et al., 2025; Levrero and Zucman-Rossi, 2016; Sidharthan and Kottilil, 2014). Against this background, our findings suggest that established HCC arising from these two etiologic contexts may retain partially distinct microbial and metabolic characteristics.

At the microbiome level, alpha diversity was broadly comparable between the two groups, whereas beta-diversity analyses suggested greater within-group heterogeneity in ALD-HCC and only modest separation between the two etiologic subgroups. Greater inter-individual microbiome dispersion has been described in several disease settings as an Anna Karenina principle–like pattern, reflecting increased stochasticity or ecological instability (Zaneveld et al., 2017; Ma, 2020). Although ALD-HCC samples appeared more dispersed, PERMDISP did not demonstrate a significant difference in multivariate dispersion; therefore, the present data do not provide sufficient evidence for a stochastic dysbiosis phenotype. The lower GMHI observed in ALD-HCC was also consistent with a microbiome profile that was less similar to health-associated reference patterns, although GMHI was not specifically developed to distinguish etiologic subgroups within HCC.

More specific taxonomic differences were observed between the two etiologic groups. LEfSe screening suggested enrichment of *Faecalibacterium*, *Roseburia*, *Lachnospira*, *Turicibacter*, and *Butyribacter* in HBV-HCC, whereas *Allisonella* and *Coprobacillus* were enriched in ALD-HCC. Importantly, some of the principal taxonomic findings were supported by the covariate-adjusted ANCOM-BC2 analysis, with *Turicibacter* and *Lachnospira* remaining FDR-supported. Several taxa enriched in HBV-HCC, including *Faecalibacterium*, *Roseburia*, and *Butyribacter*, are associated with short-chain fatty acid production (Fusco et al., 2023; Zou et al., 2021). SCFAs contribute to intestinal barrier homeostasis and immunoregulatory processes (Furusawa et al., 2013), while differences in gut microbial and metabolic profiles have also been reported across immune phases of chronic HBV infection (Li et al., 2022). These observations raise the possibility that the microbial pattern observed in HBV-HCC may reflect, at least in part, an intestinal ecological context associated with chronic HBV infection. Because SCFA concentrations and host immune phenotypes were not directly measured, however, this biological interpretation requires further validation.

The metabolomic analysis also suggested etiology-associated differences, particularly involving lipid and glycerophospholipid metabolism. This pattern has biological plausibility in the context of alcohol-related liver disease. Chronic alcohol exposure can disrupt choline and methyl-donor metabolism and impair PEMT-dependent phosphatidylcholine synthesis, potentially altering the phosphatidylcholine/phosphatidylethanolamine balance and lipoprotein export (Kharbanda et al., 2006; Li et al., 2006). At the same time, circulating phosphatidylcholine profiles are also influenced by metabolic disorders such as type 2 diabetes (Floegel et al., 2012). Because diabetes and hypertension were more frequent in the ALD-HCC group, the observed lipid-related patterns may reflect the combined effects of etiologic and metabolic factors rather than an alcohol-specific effect. Although the directions of several candidate metabolic differences were generally preserved after covariate adjustment, their statistical support was substantially attenuated after multiple-testing correction. The metabolomic and pathway findings should therefore be interpreted cautiously as exploratory observations.

The cross-omics analyses further revealed a distinction between global and local patterns of correspondence. Procrustes analysis and O2PLS indicated limited overall concordance between the microbiome and metabolome. Nevertheless, phosphatidylcholine-related features showed recurrent associations with several microbial taxa. Phosphatidylcholine is a major component of cellular membranes, and the balance between phosphatidylcholine and phosphatidylethanolamine, together with phospholipid remodeling, influences membrane integrity, biophysical properties, and lipid signaling (Li et al., 2006; Bi et al., 2024; Wang and Tontonoz, 2018). The observed PC-centered associations are therefore biologically consistent with altered membrane-lipid homeostasis. However, these correlations did not remain significant after BH correction, and tissue membrane composition or remodeling flux was not directly measured; thus, they should not be interpreted as evidence of causal microbiome-mediated membrane remodeling or coordinated gut–liver regulation.

The baseline clinical differences between the two etiologic groups also require consideration. Patients with HBV-HCC were younger, whereas diabetes and hypertension were more frequent in ALD-HCC. Such differences are clinically plausible in these etiologic populations but may themselves influence both gut microbial composition and systemic metabolism. Covariate-adjusted sensitivity analyses provided support for some of the principal microbiome and metabolomic patterns, although statistical support weakened for several individual features. Accordingly, the observed differences should be interpreted as etiology-associated subgroup patterns rather than effects attributable solely to the underlying etiology.

This study has several limitations. First, it was a single-center, cross-sectional study with a relatively small and unbalanced sample size, particularly in the ALD-HCC group. In addition, no healthy individuals or non-HCC liver-disease controls were included; therefore, the observed differences should be interpreted as contrasts between two etiologically defined HCC subgroups rather than signatures of HCC development or progression. Second, quantitative alcohol exposure, HBeAg status, HBV DNA levels, and detailed concomitant medication use were not systematically recorded, and residual clinical confounding may remain despite covariate-adjusted sensitivity analyses. Third, some specific parameters of the DADA2-based processing workflow were not described in sufficient detail, which may limit complete technical reproducibility of the 16S analysis. Fourth, the metabolomic findings showed limited support after multiple-testing correction, metabolite annotation was not based on project-specific authentic-standard confirmation, and not all microbiome candidates remained supported in the composition-aware sensitivity analysis. Finally, PICRUSt2-based functional prediction and microbiome–metabolite correlation analyses provide indirect evidence and cannot establish biological mechanisms or causal relationships. Taken together, larger multicenter studies integrating quantitative exposure assessment, metagenomic profiling, targeted quantitative metabolomics, and functional validation are needed to determine whether these candidate signals are reproducible and biologically meaningful.

## 5 Conclusion

HBV-HCC and ALD-HCC may exhibit different gut microbiome compositions, serum metabolomic features, and microbiome-metabolite association patterns. Compared with HBV-HCC, ALD-HCC appeared to exhibit greater within-group microbial heterogeneity, while *Faecalibacterium*, *Roseburia*, *Lachnospira*, *Turicibacter*, and *Butyribacter* were enriched in HBV-HCC and *Allisonella* and *Coprobacillus* were enriched in ALD-HCC. Candidate differential serum metabolic features were mainly related to lipid, bile acid, and amino acid metabolism, suggesting that different etiologic backgrounds may be accompanied by distinct gut–liver axis-related patterns in HCC. Overall, these findings should be interpreted as hypothesis-generating and require validation in larger, more deeply phenotyped cohorts.

## Data Availability

The raw 16S rRNA gene sequencing data generated in this study have been deposited in the Genome Sequence Archive (GSA) under accession number CRA043781. The untargeted metabolomics data generated in this study have been deposited in the OMIX repository under accession number OMIX017262.
