# Supplementary material for: Etiology-associated differences in the gut microbiome and metabolome in hepatocellular carcinoma: an exploratory study of alcohol-related and HBV-related cases
Source: Front Microbiol. 2026 Sep 30;17:1927595. doi: 10.3389/fmicb.2026.1927595 (PMC13671147; doi:10.3389/fmicb.2026.1927595)
Supplement: Supplementary file 1 [file Data_Sheet_1.docx]

Supplementary Material

# 1 Supplementary Figures

Figure S1. Quality control and alpha diversity assessment of gut microbiota. (A) Rarefaction curves. (B) Rank-abundance curves. (C) Alpha diversity overview.

Figure S2. Multi-level taxonomic composition of the gut microbiota. (A) Phylum-level community bar plot. (B) Class-level community bar plot. (C) Order-level community bar plot. (D) Family-level community bar plot.

Figure S3. Quality control and correlation analysis of the metabolome. (A) PCA score scatter plot of QC and study group samples. (B) PLS-DA score scatter plot of QC and study group samples. (C) Sample correlation heatmap.

Figure S4. O2PLS integration analysis of the gut microbiota and metabolome in the ALD-HCC group. (A) O2PLS joint score plot of the ALD-HCC group. (B) O2PLS loading distribution plot of the ALD-HCC group. (C) Top 10 loading species and metabolites distribution plot of the ALD-HCC group. (D) Correlation heatmap in the ALD-HCC group. ns, *P* > 0.05; *, *P* < 0.05; **, *P* < 0.01; ***, *P* < 0.001.

Figure S5. O2PLS integration analysis of the gut microbiota and metabolome in the HBV-HCC group. (A) O2PLS joint score plot of the HBV-HCC group. (B) O2PLS loading distribution plot of the HBV-HCC group. (C) Top 10 loading species and metabolites distribution plot of the HBV-HCC group. (D) Correlation heatmap in the HBV-HCC group. ns, *P* > 0.05; *, *P* < 0.05; **, *P* < 0.01; ***, *P* < 0.001.

# 2 Supplementary Tables

Table S1. Per-sample summary of processed 16S rRNA gene sequence records.

| Sample_info | Seq_num | Base_num (bp) | Mean_length (bp) | Min_length (bp) | Max_length (bp) |
| --- | --- | --- | --- | --- | --- |
| A1 | 24939 | 9726750 | 390.021653 | 110 | 536 |
| A2 | 23826 | 9390021 | 394.108159 | 131 | 533 |
| A3 | 31949 | 12586112 | 393.943848 | 94 | 536 |
| A4 | 37166 | 15177182 | 408.361998 | 204 | 424 |
| A5 | 49081 | 19209631 | 391.3863 | 129 | 423 |
| A6 | 38760 | 15433128 | 398.171517 | 105 | 536 |
| A7 | 31444 | 12475395 | 396.749618 | 171 | 535 |
| A8 | 40994 | 16798603 | 409.781992 | 240 | 447 |
| A9 | 42226 | 16512958 | 391.061384 | 98 | 535 |
| A10 | 23617 | 9529892 | 403.518313 | 73 | 535 |
| A11 | 33725 | 13507914 | 400.531179 | 173 | 423 |
| A12 | 27334 | 10926314 | 399.733446 | 132 | 536 |
| A13 | 48818 | 19050716 | 390.239584 | 131 | 433 |
| A14 | 32997 | 12901862 | 391.001061 | 131 | 536 |
| B1 | 29898 | 11799624 | 394.662653 | 131 | 536 |
| B2 | 45179 | 17606251 | 389.699883 | 93 | 536 |
| B3 | 32527 | 12955326 | 398.294525 | 103 | 536 |
| B4 | 23507 | 9345917 | 397.580168 | 131 | 536 |
| B5 | 28721 | 11389172 | 396.545106 | 131 | 535 |
| B6 | 37198 | 14796150 | 397.767353 | 131 | 536 |
| B7 | 22301 | 8748428 | 392.288597 | 131 | 536 |
| B8 | 26236 | 10228954 | 389.882375 | 194 | 535 |
| B9 | 35028 | 13656714 | 389.879925 | 106 | 535 |
| B10 | 21601 | 8460945 | 391.692283 | 78 | 536 |
| B11 | 31867 | 12645970 | 396.835912 | 103 | 537 |
| B12 | 24144 | 9532948 | 394.837144 | 238 | 435 |
| B13 | 32532 | 12776848 | 392.74708 | 112 | 537 |
| B14 | 40399 | 15691760 | 388.419515 | 131 | 421 |
| B15 | 46815 | 18416104 | 393.380412 | 110 | 536 |
| B16 | 43738 | 17486084 | 399.791577 | 65 | 517 |
| B17 | 30361 | 11933004 | 393.037252 | 85 | 536 |
| B18 | 33573 | 13154701 | 391.823817 | 103 | 417 |
| B19 | 36115 | 14165300 | 392.227606 | 186 | 535 |
| B20 | 44915 | 17801247 | 396.331894 | 80 | 536 |
| B21 | 44113 | 17128430 | 388.285313 | 101 | 535 |
| B22 | 40928 | 16239020 | 396.770426 | 153 | 421 |
| B23 | 41254 | 16209009 | 392.907573 | 119 | 414 |
| B24 | 45150 | 17949952 | 397.562614 | 155 | 536 |
| B25 | 40091 | 15820938 | 394.625677 | 68 | 535 |
| B26 | 21219 | 8366360 | 394.286253 | 161 | 436 |

Abbreviations: A, ALD-HCC group; B, HBV-HCC group; Seq_num, number of optimized sequences per sample; Base_num, total number of bases in the optimized sequences per sample; Mean_length, average sequence length per sample; Min_length, minimum sequence length per sample; Max_length, maximum sequence length per sample.

Table S2. Expression data before and after preprocessing of metabolomics sequencing.

| **Ion mode** | **All peaks** | **Identified metabolites** | **Unidentified** | **Metabolites in Library** | **Metabolites in KEGG** |
| --- | --- | --- | --- | --- | --- |
| raw-pos | 12979 | 1477 | 11502 | 1214 | 534 |
| raw-neg | 14738 | 1550 | 13188 | 1384 | 573 |
| raw-mix | 27717 | 3027 | 24690 | 2598 | 1107 |
| origin-pos | 6113 | 928 | 5185 | 760 | 334 |
| origin-neg | 8174 | 1123 | 7051 | 998 | 435 |
| origin-mix | 14287 | 2051 | 12236 | 1758 | 769 |

Abbreviation: raw-pos, raw data in positive ion mode; raw-neg, raw data in negative ion mode, raw-mix, raw data in positive and negative ion mode; origin-pos, data preprocessed in positive ion mode, origin-neg, data preprocessed in negative ion mode, origin-mix, data preprocessed in positive and negative ion modes.

Table S3. Covariate-adjusted genus-level differential abundance analysis using ANCOM-BC2.

| **Genus** | **lfc (ALD-HCC vs HBV-HCC)** | **SE** | ***P* value** | **BH-adjusted *q* value** | **Core LEfSe comparison** |
| --- | --- | --- | --- | --- | --- |
| *Lachnospiraceae_UCG-004* | −2.464 | 0.319 | 5.40 × 10^−6^ | 5.51 × 10^−4^ | — |
| *Turicibacter* | −2.263 | 0.350 | 4.61 × 10^−5^ | 0.002 | FDR-supported |
| *Limosilactobacillus* | −5.591 | 0.329 | 7.04 × 10^−5^ | 0.002 | — |
| *Agathobacter* | −2.367 | 0.547 | 6.92 × 10^−4^ | 0.010 | — |
| *CAG-56* | −2.014 | 0.327 | 8.37 × 10^−4^ | 0.010 | — |
| *Desulfovibrio* | 1.561 | 0.341 | 7.94 × 10^−4^ | 0.010 | — |
| *Dialister* | 3.607 | 0.544 | 5.68 × 10^−4^ | 0.010 | — |
| *Lachnospiraceae_ND3007_group* | −1.457 | 0.333 | 7.44 × 10^−4^ | 0.010 | — |
| *Lactococcus* | −3.104 | 0.353 | 9.25 × 10^−4^ | 0.010 | — |
| *Gemmiger* | −1.367 | 0.361 | 0.002 | 0.017 | — |
| *Tyzzerella* | −1.687 | 0.418 | 0.002 | 0.017 | — |
| *Terrisporobacter* | −1.986 | 0.348 | 0.002 | 0.020 | — |
| *[Eubacterium]_eligens_group* | −1.577 | 0.419 | 0.003 | 0.021 | — |
| *Solobacterium* | −1.591 | 0.309 | 0.004 | 0.025 | — |
| *UCG-002* | −1.527 | 0.414 | 0.004 | 0.025 | — |
| *Parasutterella* | −1.565 | 0.313 | 0.004 | 0.026 | — |
| *Raoultibacter* | −1.831 | 0.315 | 0.004 | 0.026 | — |
| *Megasphaera* | −2.071 | 0.371 | 0.005 | 0.029 | — |
| *[Eubacterium]_ventriosum_group* | 1.296 | 0.390 | 0.006 | 0.030 | — |
| *Ligilactobacillus* | −1.654 | 0.450 | 0.006 | 0.031 | — |
| *Ruminococcus* | −1.833 | 0.584 | 0.006 | 0.031 | — |
| *Intestinibacter* | −1.451 | 0.457 | 0.007 | 0.034 | — |
| *Schaalia* | −1.061 | 0.359 | 0.010 | 0.046 | — |
| *Lachnospira* | −1.441 | 0.525 | 0.011 | 0.047 | FDR-supported |
| *Blautia* | −1.443 | 0.541 | 0.012 | 0.048 | — |
| *Faecalibacterium* | −0.890 | 0.675 | 0.199 | 0.366 | Direction-concordant |
| *Roseburia* | −0.064 | 0.614 | 0.918 | 0.936 | Direction-concordant |

Note: ANCOM-BC2 models were adjusted for age, BMI, diabetes, and hypertension. The lfc represents the ANCOM-BC2 log-fold-change estimate for ALD-HCC relative to HBV-HCC; positive values indicate higher abundance in ALD-HCC and negative values indicate higher abundance in HBV-HCC. *P* values are unadjusted, and q values are BH-adjusted. “FDR-supported” indicates a core LEfSe genus with BH-adjusted q < 0.05, whereas “Direction-concordant” indicates a core LEfSe genus that retained the original direction but did not remain significant after BH correction.
